# Metabolic Rates Predict Baseline Corticosterone and Reproductive Output in a Free-Living Passerine

**DOI:** 10.1093/iob/obaa030

**Published:** 2020-10-14

**Authors:** Blanca Jimeno, Mackenzie R Prichard, Devin Landry, Cole Wolf, Beau Larkin, Zachary Cheviron, Creagh Breuner

**Affiliations:** 1 Division of Biological Sciences, University of Montana, Missoula, MT 59812, USA; 2 Department of Psychology, Emory University, Atlanta, GA 30322, USA; 3 MPG Ranch, Florence, MT 59833, USA

## Abstract

Organisms continuously face environmental fluctuations, and allocation of metabolic investment to meet changing energetic demands is fundamental to survival and reproductive success. Glucocorticoid (GC) hormones (e.g., corticosterone [CORT]) play an important role in energy acquisition and allocation in the face of environmental challenges, partly through mediation of energy metabolism. Although GCs and metabolic rate are expected to covary, surprisingly few empirical studies have demonstrated such relationships, especially in wild animals. Moreover, studies testing for associations between GCs and fitness generally do not account for among-individual differences in energy expenditure or energy allocation. We measured CORT (baseline and stress-induced) and metabolic traits (resting metabolic rate [RMR], cold-induced VO_2max_ [M_sum_], and aerobic scope [the difference between M_sum_ and RMR]) in female tree swallows (*Tachycineta bicolor*) during chick-rearing, and tested for their associations with several variables of reproductive performance. We found a positive relationship between RMR and baseline CORT, but no consistent associations between stress-induced CORT (SI-CORT) and M_sum_. This suggests that while baseline CORT may be a good indicator of an individual’s baseline metabolic investment, SI-CORT responses are not associated with aerobic scope or the upper limits of aerobic performance. Furthermore, we found that metabolic traits were associated with reproductive performance: females with higher reproductive output showed higher M_sum_, and also tended to show higher RMR. Overall, these results suggest that metabolic traits are better predictors of reproductive output in tree swallows than CORT concentrations. They further point to the maximal aerobic capacity being higher in females investing more heavily in a current reproductive event, but whether this association reflects trade-offs between current and future reproductive efforts remains to be tested.

## Abstract (es)

Los organismos se enfrentan continuamente a fluctuaciones ambientales, y la distribución de la inversión metabólica es fundamental para la supervivencia y el éxito reproductivo ante demandas energéticas cambiantes. Las hormonas glucocorticoides (GC), como la corticosterona (CORT), juegan un papel importante en los procesos de adquisición y distribución de energía durante los desafíos ambientales, en parte a través de su papel mediador del metabolismo energético. Aunque es esperable que los GCs y la tasa metabólica covaríen, muy pocos estudios empíricos han demostrado esta relación, especialmente en animales salvajes. Además, los estudios que han testado la asociación entre GCs y eficacia biológica normalmente no tienen en cuenta las diferencias en gasto o distribución energética existentes entre individuos. En este estudio, medimos CORT (basal e inducida por estrés) y parámetros metabólicos [tasa metabólica en reposo (RMR), VO_2max_ inducido por frío (M_sum_) y el alcance metabólico (la diferencia entre M_sum_ y RMR)] en hembras de golondrina bicolor (*Tachycineta bicolor*) durante el período de crianza de los pollos, y testamos sus asociaciones con varios parámetros reproductivos. Encontramos una relación positiva entre RMR y CORT basal, pero ninguna asociación consistente entre CORT inducida por el estrés y M_sum_. Esto sugiere que mientras que la CORT basal sería un buen indicador individual del gasto metabólico base, las respuestas de CORT inducidas por estrés no están asociadas con el alcance metabólico o con los límites superiores del rendimiento aeróbico. Encontramos, además, una asociación entre parámetros metabólicos y reproducción: las hembras con mayor éxito reproductivo mostraron mayor M_sum_, y una tendencia a mostrar mayor RMR. A nivel general, estos resultados sugieren que los parámetros metabólicos son mejores predictores del éxito reproductivo que los niveles de CORT en la golondrina bicolor. También apuntan a que la capacidad aeróbica máxima es mayor en aquellas hembras que invierten más intensamente en el evento reproductivo actual, pero queda por investigar si esta asociación refleja compromisos entre esfuerzo reproductivo actual y futuro.

## Introduction

Organisms continuously adjust their physiology and behavior to maintain homeostasis in the face of environmental fluctuations. Environmental changes are often associated with changes in energetic demands, which makes allocation of metabolic investment of fundamental importance to survival and reproductive success (Burton et al. 2011; Welcker et al. 2015). Indeed, energy is often thought to be a limited resource, and animals are required to allocate it among competing life-history traits to maximize fitness (Stearns 1992; Welcker et al. 2015; Williams 2018). Resting metabolic rate (RMR) represents the minimal metabolism of an individual in a relatively quiescent state and is defined as the lowest metabolic rate of an endotherm while it is resting in its thermoneutral zone. It differs from the more familiar basal metabolic rate that does not require that individuals are postabsorptive and nonreproductive (Careau et al. 2015). The remaining energy expended will always be within the energetic scope set by its maximum metabolic rate, which is often referred to as VO_2max_ and represents the maximum rate of oxygen consumption (Burton et al. 2011; reviewed in Careau et al. 2015). Variation in these metabolic traits is heritable (Nilsson et al. 2009; Wone et al. 2009), and generally repeatable through time (Nespolo and Franco 2007; Broggi et al. 2009; White et al. 2013), but they are also strongly influenced by environmental variation, even after correcting for the effects of size, age, and sex (Burton et al. 2011; Cheviron et al. 2013). The rate at which an animal expends energy during a life-history event will be determined by the relative amount of energy allocated among different requirements (e.g., self-maintenance vs. reproduction) and is expected to have an impact on fitness prospects; Welcker et al. 2015).

The allocation of metabolic resources is determined by many physiological processes. Glucocorticoid (GC) hormones (e.g., cortisol, corticosterone [CORT]) play a key role in energy balance in response to environmental challenges, by mediating physiological processes involved in energy metabolism (Astheimer 1992; McEwen and Wingfield 2003; Francis et al. 2018). From baseline concentrations in an undisturbed animal, GC levels increase during periods of high energy demand, such as under low ambient temperatures (Jimeno et al. 2018c; Crino et al. 2020) or when caring for offspring (Bonier et al. 2009, 2011; Ouyang et al. 2011a). GCs are also involved in the responses to unpredicted events or threats, with plasma concentrations typically increasing (“stress response”) within a few minutes of exposure to a noxious stimulus (O’Reilly and Wingfield 2001; Romero and Reed 2005). These increases are thought to mediate physiological and behavioral responses to promote survival functions at the expense of non-essential processes, such as growth and reproduction (Wingfield et al. 1983; Ouyang et al. 2012). Thus, GCs are thought to fluctuate with anticipated or unpredicted energetic needs, and are considered indicators of physiological stress associated with metabolic investment (Kitaysky et al. 2010). Therefore, metabolism and GC concentrations are generally assumed to covary (McEwen and Wingfield 2003; Romero et al. 2009; Francis et al. 2018). Despite this expectation, there are surprisingly few empirical tests of the relationships between GC concentrations and metabolism (e.g., Haase et al. 2016; Francis et al. 2018; Jimeno et al. 2018b). Among the tests that do exist in an ecological context, among- and within-individual differences in metabolic rate and GCs are often ignored. Furthermore, while a close association between GCs and metabolism is often predicted for baseline levels, the expected relationship between stress-induced GCs and maximum metabolic rates is less clear (see Haase et al. 2016; Francis et al. 2018). The main reason to expect an association between induced increases in metabolic rates and GC levels is that GCs mobilize glucose to face an increase in metabolic demands (Jimeno et al. 2018b), and therefore the magnitude of an acute metabolic response may then be comparable to the magnitude of an acute GC response.

Reproduction is considered an energetically demanding life-history stage (Clutton-Brock 1991; Stearns 1992; Piersma and Van Gils 2011; but see Williams 2018). Daily energy expenditure often peaks during the period of rearing young (Welcker et al. 2015), and measurements in free-living great tits (*Parus major*), European starlings (*Sturnus vulgaris*), and captive zebra finches (*Taeniopygia guttata*) have shown that reproductive females have a RMR 22–27% above non-reproductive values (Nilsson and Råberg 2001; Vézina and Williams 2002, 2005). Hence, the amount of energy that an animal dedicates to a given reproductive bout is a central resource allocation decision that may have important repercussions for their current and future reproductive performance and survival. While metabolic rates and reproductive effort are expected to covary, the mechanistic underpinnings of these correlations are not clear, especially for baseline metabolism. Higher baseline metabolism may be positively correlated with fitness if it reflects a greater investment in “metabolic machinery” that facilitates greater aerobic performance but necessitates higher resource intake rates (Biro and Stamps 2010). Under this “increased intake” hypothesis, higher baseline metabolism is expected to have a positive effect on fitness (Mueller and Diamond 2001; Burton et al. 2011; reviewed in Careau et al. 2015 ). However, elevating baseline metabolism may be energetically expensive (Nagy et al. 1999), and a lower baseline energy expenditure may improve fitness because excess energetic resources could be redirected to other functions such as reproduction. This “compensation” hypothesis (Nilsson 2002) predicts that baseline metabolism will be negatively correlated with fitness (reviewed in Burton et al. 2011; Careau et al. 2015). Taking a comparable framework, if baseline GCs are related to metabolic investment, it could be argued that higher baseline GC concentrations during the breeding season may be associated with increased reproductive success (“CORT-adaptation hypothesis”; Chastel et al. 2005; Bonier et al. 2009, 2011). In contrast, elevated GC levels are often thought to have a negative impact on reproduction (“CORT-tradeoff hypothesis”; Greenberg and Wingfield 1987; Patterson et al. 2014; Breuner and Berk 2019). Surprisingly, and despite the assumed relationship between GCs and metabolism, studies testing for associations between GCs (either baseline or stress-induced levels) and fitness generally do not account for among-individual differences in energy expenditure or energy allocation. Thus, energetic needs and fluctuations in metabolic rates may underlie GC variation, which can make the relationships between GCs (or metabolic rates) and fitness context-dependent (e.g., as a function of resource availability; Burton et al. 2011; Schoenle et al. 2018). This adds to the fact that if metabolic needs mediate the effects of GC on fitness, we may expect weaker correlations when testing the associations between GCs and fitness, compared with metabolic rates and fitness. Furthermore, while elevated M_sum_ (a cold-induced VO_2max_ and proxy for maximal metabolic output) and higher aerobic scopes have been shown to be associated with enhanced survival in some contexts (Hayes and O'Conner 1999; Petit et al. 2017), it remains unclear whether higher values in the acute GC responses reflect better fitness prospects (Moore and Hopkins 2009; Breuner et al. 2008; Vitousek et al. 2018; Zimmer et al. 2019).

We measured metabolic rates (RMR, M_sum_, and aerobic scope [the difference between M_sum_ and RMR]) and CORT concentrations (baseline, stress-induced, and the associated increase [the difference between stress-induced and baseline]) in breeding females of two wild tree-swallow (*Tachycineta bicolor*) populations in western Montana (USA). We then tested for associations between CORT concentrations and metabolic rates and reproductive output. We predicted that (1) there will be an association between resting metabolism and baseline CORT traits, with higher CORT concentrations reflecting higher energy expenditure; (2) metabolic traits during the breeding season will be associated with reproductive output; and (3) If CORT reflects metabolism, the associations between CORT and metabolic rate with reproductive output should be in the same direction, but may be weaker for CORT traits. The latter would be due to GC variation being a byproduct of the variation in energy expenditure needed to fulfill the energetic demands of reproduction. Weaker associations for CORT traits may also be expected due to lability and high level of variation in GCs compared to metabolic rates.

## Materials and methods

### Study population

Use of animals for this research was approved by the Institutional Animal Care and Use Committees at the University of Montana (protocol number AUP 014-16CBOBE-072116). We measured metabolic and CORT traits in two populations of tree swallows (*N* = 26) in Western Montana over two breeding seasons (March through July 2016 and 2017): MPG North (47°31′28″N/113°40′28″W; *N* = 10 in 2016; *N* = 2 in 2017) and MPG Ranch (46°40′07″N/114°01′22″W; *N* = 14 in 2017).

Tree swallows are obligate secondary cavity nesters that readily use artificial nest boxes. Nests were checked twice weekly at the beginning of the season, at least every other day after the first egg was laid, twice a week through incubation, and every other day through the nestling growth period. We stopped weighing nestlings before Day 15 to avoid premature fledge. On Day 3 of incubation females were captured, banded, and measured, and the outer two primaries marked along the bottom ∼2 cm with White-Out to enable ID during nest videos (see below). Females were captured inside the nest box, either by covering the entrance hole when we arrived at the box (often possible for the first capture), or by using a trap activated when female entered. The same females were captured again between post-hatching day (PHD) 11–13 for blood sampling and metabolic measures. Our dataset therefore only includes females that succeeded up to this date. On first capture, females were banded with a nine digit ID ring and a bicolor plastic ring. We measured the head-bill, wing chord, tarsus, and weight of each female at both captures, and the same measures were taken from nestlings every 2–3 days post-hatch until Day 13–15, as part of our field protocol. Female structural size was measured as the average tarsus, wing chord, and head + bill length after each measure was transformed to a standard normal distribution (Briga et al. 2017). Residual body mass was calculated as the residuals of the linear regression of body mass on structural size, to obtain a mass component independent of size. In this study, we collected information on several reproductive variables including number of hatchlings, number of PHD 12–13 nestlings (which in our population is a good indicator of fledgling success as survival rate from Day 13 until fledge is >95%), feeding rate, and average nestling mass (calculated by dividing the total nestling mass by nest at PHD 12–13 by the brood size), as an indicator of the amount of energy/resources brought into the nest per chick up to that date.

Female feeding rates were recorded with Veho Muvi MicroDV Camcorders (VCC-003-MUVI) Velcroed inside the nest boxes. Feeding visits were recorded for up to 2 h and corrected to feeding rate/hour given total video time. Average recording start time was 10:05 am ± 6 min SEM, with a range of 7:40–12:40. These data were collected in late June/early July, so the first bout of feeding as the sun comes up (∼5:30–6:00) is well over. Videos were not taken if it was raining or during high winds (conditions significantly affecting insect availability). Videos were recorded between 5 and 11 PHD (7.3 ± 0.48, mean ± SEM), and there was no effect of nest age on feeding rate (estimate = −0.07, *P* = 0.59, *R*^2^ = 0.01).

### CORT sampling

CORT samples were taken between 11 and 13 PHD, and females were caught and sampled in the late afternoon. Blood samples were collected into heparinized microhematocrit tubes from the alar vein after venipuncture with a 30 G needle. Three serial samples were taken from each female: once baseline CORT (herein, BasCORT) sample within 3 min of capture, and the two stress-induced CORT (herein, SI-CORT) samples 10 and 30 min after capture; birds were held in a small cloth bag between samples (as in Wingfield et al. 1992). One microhematocrit tube of blood (∼60 uL) was collected at each sampling. Blood was kept on ice for up to 4 h, then centrifuged and the plasma removed and frozen until assayed.

### CORT assay

CORT levels were measured using Enzo Life Sciences EIA kit (cat #901-097) as per Patterson et al. (2011). Briefly, 2000 riefly, 20001“ \o ”51=Ref Patterson SH, WinCORT was extracted from 20 uL of plasma through a double ether extraction. Samples were run in duplicate or triplicate as plate space allowed, at a 1:20 dilution. Samples from the same individual were included in the same plate. Resulting values were corrected to 100% recovery values based on tracer left in sample after extraction (2016: 51 ± 1.5%; 2017: 71 ± 2.2%). Samples were run across two plates in 2016 and five plates in 2017. Mean intra-assay CofV across all plates was 8%, inter-assay CofV was 1% in 2016, and 16% in 2017; the level of detectability averaged 0.6 pg/100 uL, with only one sample reading below detectability. That sample was set to the level determined for that plate (0.5 pg/100 uL).

### Metabolic rate measurements

Metabolic rates were taken at the same capture as the CORT samples. After the blood samples were taken, females were given Gatorade and held in cages with seed, water, and oranges until MR sampling. We measured RMR and cold-induced VO2max (summit metabolic rate—M_sum_) using open-flow respirometry in wild-caught females following standard protocols. RMR trials were conducted during the rest phase between 18:00 and 23:00 h, and M_sum_ trials conducted the following morning between 08:00 and 11:00 h. Following these metabolic measurements, birds were immediately returned to their site of capture and released, and the experimental protocol did not result in nest abandonment by the female in any case. All metabolic measurements were taken within 3 weeks (June 25–July 12), and there was no effect of date (*F*_1,21_= 0.48, *P* = 0.50) or the time of day (*F*_1,21_= 0.14, *P* = 0.71) on RMR.

For the RMR measurement, birds were placed in 1 L respirometry chamber placed inside of a temperature cabinet (Sable Systems Pelt Cabinet with Pelt-5 Temperature Controller). All RMR trials were performed at 27°C, which is within the thermoneutral zone for similarly sized passerine birds (Pollock et al. 2019). We measured up to three individuals simultaneously by cycling through measurements on focal individuals at 15-min intervals, and the measurement on the first individual began immediately after it was placed in the chamber. After cycling through individual animals in this manner we performed a 15-min ambient baseline measurement using an empty chamber that was identical to the animal chambers. With this cycling protocol, each individual was measured for at least 45 min over the course of 3 h trial, and RMR was typically achieved during the second or third cycle. Incurrent air was dried with Drierite and pumped through the animal chamber at rate of 500 mL/min. Excurrent air from the animal and baseline chambers was subsampled and redried with Drierite before being passed through a CO2 analyzer in a FoxBox (Sable Systems). Following the CO2 measurement, CO2 was scrubbed with Ascarite and the outflow dried again with Drierite before passing through the FoxBox O2 analyzer. Prior to each day’s measurements, we spanned the O2 analyzer FoxBox using ambient air at ∼20.95% O_2_. We quantified rate of instantaneous oxygen consumption following Lighton (2008) and corrected for analyzer drift using the baseline measurements. We defined RMR as the lowest rate of oxygen consumption (mL O2/min) averaged over a 10-min sliding window using custom scripts in the R programming environment. RMR was calculated in windows with steady gas traces, indicating the individual was calm and at rest. Because we did not record the time at which individuals were captured or whether they consumed food during captivity, we cannot be sure that individuals were postabsorptive at the beginning of the RMR trials, but they likely were at the end.

M_sum_ is a cold-induced measure of VO_2max_ that can serve as a proxy for an individual’s maximal rate of oxygen consumption (Zhang et al. 2015). While not a direct measure of exercise-induced maximal metabolic rate (MMR), cold-induced M_sum_ has been shown to exceed MMR measured using common approaches such as hop-flutter wheels in a closely related congener (*T. albilinea*, Wiersma et al. 2007), suggesting that M_sum_ is more effective than hop-flutter wheels for inducing maximal rates of oxygen consumption in swallows. We performed the M_sum_ trials using a heliox (21% helium, 79% oxygen) atmosphere. The high thermal conductance of heliox facilitates heat loss at higher temperatures than air, allowing for the elicitation of VO_2max_ without risk of cold injury (Rosenmann and Morrison 1974). All trials were conducted at a static temperature of −4°C, and heliox flow rates of 750 mL/min. Heliox flow rates were measured using Alicat M-series flow meters calibrated for heliox. Trials ended when CO2 production or O2 consumption declined for 5 min. M_sum_ was defined as the highest oxygen consumption (mL O2/min) averaged over a 5-min sliding window using custom scripts in R. Most individuals reached M_sum_ within 10–20 min of the trial start. We measured body temperature (T_b_) before and after the M_sum_ trial by inserting a thermistor probe into the cloaca. All measured individuals were hypothemic (T_b_ < 35°C) following the M_sum_ measurements. Again, we followed Lighton (2008) to calculate rates of instantaneous oxygen consumption, and we corrected for drift using baseline measurements. Aerobic scope was calculated as the difference between M_sum_ and RMR.

### Statistical analyses

Reproductive output variables were reduced with a principal component (PC) analysis (Supplementary Fig. S1), using the data from those females captured in the two populations in 2016 and 2017 for which all reproductive variables had been measured (*N* = 22). This analysis resulted in four PCs, with PC4 explaining only ∼1% of the variance and PC3 (∼13%) not having a clear biological interpretation. The first PC (PC1) explained 63% of variation (eigenvalue = 2.52) and had factor loadings with a positive tendency for number of hatchlings (0.75), number of PHD 12–13 nestlings (0.96), and average PHD 12–13 nestling mass (0.94; Supplementary Fig. S1). Thus, higher values of PC1 were associated with larger, heavier broods with high survival until PHD 13. We interpret PC1 as higher nest success. The second PC (PC2) explained 22.6% of variation (eigenvalue = 0.91) and showed negative values for higher feeding rates (−0.91; Supplementary Fig. S1), that is, females with lower PC2 values visited the nest more. We interpret PC2 as feeding investment during chick rearing. For clarity and interpretation reasons we changed the sign of PC2, so that higher PC2 values were associated with higher feeding rates, and therefore higher values of both PCs represent higher reproductive investment. These two first components explained 85.6% of variance and were included as dependent variables in the statistical models testing for the relationships between metabolic/CORT traits and reproduction.

To test for correlations among CORT concentrations, metabolic traits, and reproduction, we constructed three series of linear models, with the 26 individuals for whom we took CORT and metabolic measurements. Final sample sizes differed by variable (Supplementary Table S1), as we could not obtain all variables for every individual (CORT, metabolic, and reproduction PCs). We tested for the association between: (1) CORT concentrations and metabolic rate, where we included CORT traits as dependent variables in three models testing for the association between BasCORT and RMR (*N* = 21), SI-CORT and M_sum_ (*N* = 22), and stress-induced increase in CORT and aerobic scope (*N* = 19); (2) CORT concentrations and reproductive output (*N* = 18); and (3) metabolic traits and reproductive output (*N* = 18). (2) and (3) included PC1 or PC2 (reproduction) as the dependent variable, as a function of either metabolic or CORT traits. Besides the main metabolic or CORT predictor, we included structural size and residual body mass as covariates, to correct for their association with metabolic rate (Schmidt-Nielsen 1984) and CORT (Lendvai et al. 2014; Jimeno et al. 2018b). We also included population as categorical factor, to control for the differences between our two sampling sites. Finally, we also included the interaction between population and the main predictor in each model (i.e., metabolic or CORT variable) to test for potential differences in the association between metabolic rate, CORT, and reproduction in our two populations. We used Akaike’s Information Criterion with correction for small sample sizes (AICc; Akaike 1974) to identify the models best supported by the data, for which a change in AICc of 2 is considered significant (Burnham et al. 2011). We selected those models within ▵AICc < 2 as best fitting models, and further explored the effects of the main predictors (i.e., metabolic or CORT variables) when present in these top models.

We added the second population in 2017 because MPG North did not provide a large enough sample size (*N* = 2); MPG North was mostly sampled in 2016, and MPG Ranch was only sampled in 2017. As a consequence, we cannot reliably separate the effects of year from the effects of population, or aim to detect differences among years within populations. We did however include population as predictor throughout. The strong population effect, when compared to the effect of year, was further supported by (1) models including population having always higher *R*^2^ and lower AICc compared to those including year instead and (2) the two individuals from the north sampled in 2017 for which both RMR and BasCORT values were available to fit into the range by population—not by year—in these variables (the ones that differed the most between the two groups; Supplementary Fig. S2).

All statistical analyses were performed using R version 3.6.1 (R Core Team 2019). PCA analyses were performed using the *prcomp* function with scaled variables. Logarithmic transformations (ln) were performed to normalize CORT variables in the analyses. CORT increases were calculated as ln(SI-CORT) – ln(BasCORT). Pearson correlations were also obtained to test the association between metabolic, CORT, and reproductive variables (see Supplementary Table S2). We used Type III sum of squares with the *Anova* function in the *car* package (Fox and Weisberg 2019) because it is the most accurate when the experimental design is unbalanced. After model selection, we checked for normality of residuals, heteroskedasticity, and multicollinearity using the *check_model* function of the package *performance* (Lüdecke et al. 2020). While building the models including BasCORT, one individual female was excluded from the analyses because it was a statistical outlier (sampling time for BasCORT exceeded the 3 min threshold, and this data point was >2 times the standard deviation of the model residuals). Graphs were generated using the *ggplot2* package (Wickham 2016).

## Results

### GC traits and metabolic traits

**Fig. 1:**
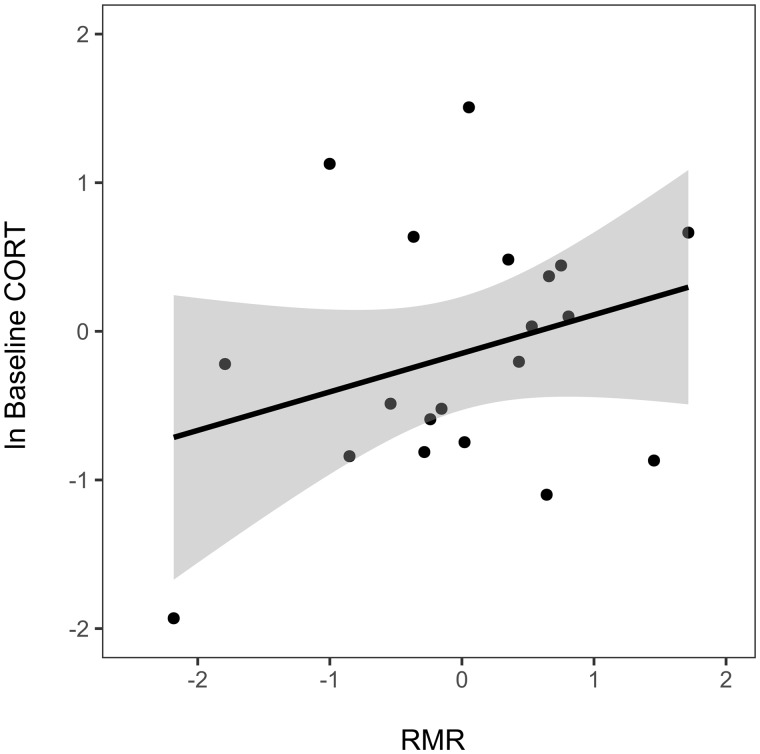
Relationship between RMR and BasCORT. Note that both variables were standard-normalized by population (*x*-mean/SD) for visualization purposes.

The best fitting model explaining the variation in BasCORT included RMR, population, and size-corrected body mass as predictors (Table 1). We found a positive association between BasCORT and RMR (*F*_1,16_=4.60; *P* < 0.05; Table 1, Fig. 1). This model also showed a strong association between BasCORT and residual body mass, with heavier females showing lower BasCORT concentrations (*F*_1,16_=10.68, *P* < 0.01; Table 1, Supplementary Fig. S3).

**Table 1:** Model comparisons showing the best fitting models (▵AICc < 2) predicting baseline CORT (ln)

BasCORT (ln)	*K*	logLik	AICc	▵AICc	Weight
RMR, body mass, population	4	2.12	10.0	0.00	0.48
Body mass, population	3	−0.41	11.5	1.44	0.23
	**Estimate**	**SE**	**df**	***F*-value**	***P*-value**
Intercept	1.36	0.22	16 (1)		
RMR	0.40	0.19	16 (1)	4.60	<0.05
Body mass	−0.18	0.05	16 (1)	10.68	<0.01
Population	−1.74	0.17	16 (1)	110.85	<0.001

**Fig. 2:**
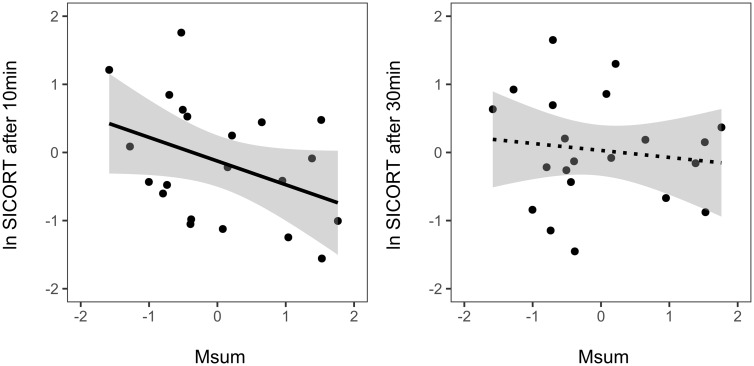
Relationship between M_sum_ and SI-CORT after 10 min (**A**) or 30 min (**B**) of restraint. Dotted lines represent non-significant associations. Note that variables were standard-normalized by population (*x*-mean/SD) for visualization purposes.

There were some differences between the best fitting models explaining the variation in SI-CORT after 10 or 30 min of restraint, and including M_sum_ as predictor. The best model for SI-CORT 10 (and only model within ▵AICc < 2) included only M_sum_ as predictor (Supplementary Table S3a). This model showed a negative and significant relationship between SI-CORT after 10 min and M_sum_ (*F*_1,20_=10.64, *P* < 0.01, Supplementary Table S3a, Fig. 2A). In contrast, model selection for SI-CORT 30 supported three models within ▵AICc < 2. Only one of these models (▵AICc = 1.15) included M_sum_ as predictor, together with population (Supplementary Table S3b), but the association between M_sum_ and SI-CORT 30 was not significant (Supplementary Table S3b, Fig. 2B). Aerobic scope was included, together with population and residual body mass, in one of the models best explaining the variation in CORT increase after 10 min of restraint (▵AICc = 1.41; Supplementary Table S4a). The relationship between aerobic scope and CORT increase was not significant (*F*_1,15_=1.97, *P* = 0.18; Supplementary Table S4a), and was negative as the one found between SI-CORT 10 and M_sum_, likely due to the mathematical dependence of M_sum_ and aerobic scope and SI-CORT and CORT increase, respectively. In contrast, aerobic scope was not included in any of the models best explaining the variation of CORT increase after 30 min of restraint, which included population and residual body mass only (Supplementary Table S4b).

### GCs, metabolism, and reproductive output

**Fig. 3:**
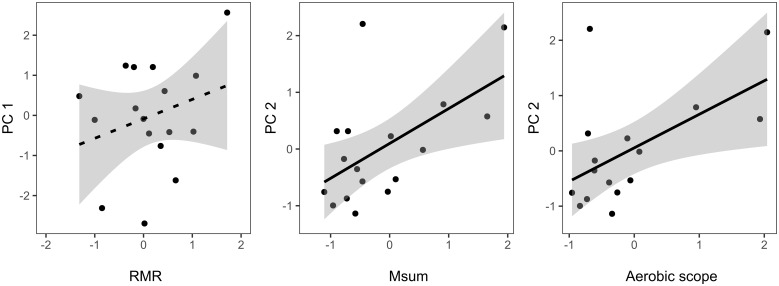
Relationship between metabolic traits and reproductive output (PC1 and PC2). Dotted lines represent non-significant associations. Note that metabolic variables were standard-normalized by population (*x* mean/SD) for visualization purposes.

Among the selected models (▵AICc < 2) best explaining variation in PC1 (i.e., including either metabolic or CORT predictors; Tables 2, Supplementary Tables S5–S8), only two included a metabolic or CORT predictor: RMR (▵AICc = 0.07; Table 2) and BasCORT (▵AICc = 1.73; Supplementary Table S5). The model including RMR did not include any other variable, but the association between PC1 and RMR was not significant (Supplementary Table S5, Fig. 3). The model including BasCORT also included population, and the interaction between population and BasCORT, which was significant (*F*_1,13_= 6.82, *P* = 0.02). To explore this interaction, we ran separate linear models for the two populations (MPG Ranch and MPG North), and we found a trend for a positive association between BasCORT and nest success in MPG North (Estimate= 5.32 ± 2.40, *F*_1,7_= 4.90, *P* = 0.06), but not in MPG Ranch (Estimate= 0.06 ± 2.30, *F*_1,6_= 0.04, *P* = 0.85).

**Table 2:** Model comparisons showing the best fitting models (▵AICc < 2) predicting reproductive output (PC1) and including RMR as predictor

PC1	*K*	logLik	AICc	▵AICc	Weight
Null	1	−28.66	62.2	0.00	0.23
RMR	2	−27.20	62.3	0.07	0.22
Population	2	−27.88	63.6	1.41	0.11
	**Estimate**	**SE**	**df**	***F*-value**	***P*-value**
Intercept	−1.94	1.18	15 (1)		
RMR	1.44	0.86	15 (1)	2.81	0.11

Among the selected models (▵AICc < 2) best explaining variation in PC2 (i.e., including either metabolic or CORT predictors; Table 3, Supplementary Tables S9–S12), three models included a metabolic or CORT predictor: M_sum_ (▵AICc = 0.00; Table 3), Aerobic scope (▵AICc = 0.00; Table 3), and SI-CORT 10 (▵AICc = 1.85; Supplementary Table S11). Model parameters showed a positive and significant association between PC2 and both M_sum_ (*F*_1,15_= 7.54, *P* = 0.02; Table 3) and aerobic scope (*F*_1,15_= 7.86, *P* = 0.01; Table 3). Thus, higher feeding investment was correlated with higher M_sum_ and aerobic scope (Fig. 3), whereas the association between SI-CORT 10 (▵AICc = 1.85) and PC2 was not significant (Supplementary Table S11).

**Table 3:** Model comparisons showing the best fitting models (▵AICc < 2) predicting reproductive output (PC2) and including M_sum_ and aerobic scope as predictors

PC2	*K*	logLik	AICc	▵AICc	Weight
M_sum_	2	−19.94	47.7	0.00	0.44
M_sum_, population	3	−19.14	49.6	1.89	0.17

	**Estimate**	**SE**	**df**	***F*-value**	***P*-value**

Intercept	−1.76	0.68	15 (1)		
M_sum_	0.35	0.13	15 (1)	7.54	0.02

**PC2**	***K***	**logLik**	**AICc**	**▵AICc**	**Weight**

Scope	2	−18.90	45.8	0.00	0.49

	**Estimate**	**SE**	**df**	***F*-value**	***P*-value**

Intercept	−1.42	0.55	14 (1)		
Scope	0.38	0.13	14 (1)	7.86	0.01

## Discussion

In this study, we tested for the associations between metabolic rate, CORT concentrations and reproductive output in free-living, breeding female tree swallows. Among the best fitting models explaining the variation of CORT traits, we found a positive relationship between RMR and BasCORT, and a negative relationship between M_sum_ and SI-CORT after 10 min., but not after 30 min (see below). We also found that variation in metabolic traits, rather than CORT traits, explained variation in reproductive performance, with higher feeding investment (PC2) being strongly associated with higher M_sum_ and higher aerobic scope. We also found a positive association between BasCORT and nest success (PC1), but only in one of our populations, MPG North. Moreover, we found a trend toward a positive association between nest success (PC1) and RMR, although these associations were not significant.

The positive association between RMR and BasCORT suggests that BasCORT is a good indicator of an individual’s baseline metabolic investment. This result is supported by previous evidence of a positive relationship between BasCORT and environmental factors associated with increased energy expenditure, such as parental investment or ambient temperature (Welcker et al. 2015; Bauch et al. 2016; Jessop et al. 2016; Jimeno et al. 2018c). In contrast, the negative association that we found between SI-CORT after 10 min of restraint and M_sum_ and between CORT increase after 10 min and aerobic scope suggests that elevated SI-CORT levels do not reflect an enhanced capacity for aerobic performance in our populations. Quite the opposite, individuals with higher upper limits of aerobic performance and higher aerobic scopes had lower SI-CORT levels after 10 min of restraint and lower associated CORT increases. However, the fact that we found a relationship between M_sum_ and SI-CORT after 10 min, but not between M_sum_ and SI-CORT after 30 min, suggests that females with higher M_sum_ may exhibit slower increases, but not lower maximum CORT levels in responses to restraint (see Lipowska et al. (2019). Indeed, the fact that the speed of the response will highly determine the measurements of SI-CORT, whereas M_sum_ is measured as an absolute value induced by cold, could be masking potential correlations between parallel increases in metabolic rate and CORT. Thus, the two types of measures may co-vary in real time, but not covary has taken given the different timing of assessment. Moreover, while RMR and BasCORT are assumed to reflect the same physiological state, this does not necessarily hold for SI-CORT when compared with M_sum_. SI-CORT response may be stimulus-specific (Canoine et al. 2002), and reflect the change in metabolic rate induced by that stimulus (Jimeno et al. 2018b). It is therefore possible that the restraint protocol did not induce maximum response of the same magnitude as the cold treatment used to obtain M_sum_. Indeed, ACTH-induced CORT levels—which are assumed to reflect the maximum CORT release capacity and are often higher than stress-induced levels (Schmidt et al. 2014; Jimeno et al. 2018a)—may show a stronger correlation with M_sum_ and aerobic scope, respectively; but this prediction remains to be tested.

Our findings partially contrast with previous studies finding no overall relationship between metabolic rate and GCs in birds (e.g., Buehler et al. 2012; Francis et al. 2018). It is important to note, however, that these studies did not measure metabolic and CORT variables in the same individuals (e.g., Francis et al. 2018), or else metabolic and hormone measurements were spaced in time (e.g., Buehler et al. 2012). Indeed, a previous study including within-individual, simultaneous measurements of metabolic rate and GCs found consistent relationships between metabolic rate and CORT levels across stimuli (Jimeno et al. 2018b). Our results complement this finding suggesting that BasCORT is a reliable indicator of baseline metabolic expenditure in free-living birds, and highlight the importance of studying within-individual variation to obtain reliable patterns concerning the relationships between GCs and metabolism.

We found significant associations between metabolic traits and reproductive performance. Higher feeding investment (PC2) was correlated with higher M_sum_ and aerobic scope, while there was a trend for higher nest success (PC1) being associated with higher RMR, although the association was not significant, probably due to our modest sample size. A positive relationship between metabolic traits (RMR) and reproductive output would be in line with the “increased-intake” hypothesis, indicating a greater energetic investment to support reproductive efforts. The strong and positive relationship between aerobic scope and feeding investment (PC2) is likely explained by the positive relationship between reproductive output and M_sum_, because aerobic scope is defined as the difference between M_sum_ and RMR and it is mathematically dependent on M_sum_. This positive association between M_sum_ and feeding investment (PC2) may reflect a training effect of the increased workload associated with higher provisioning rates and fits a general pattern previously reported in vertebrates, showing positive relationship between aerobic capacity and greater endurance at submaximal activities (Bennett 1991). In house sparrows (*Passer domesticus*), exercise training elevates both maximal aerobic capacity and M_sum_ (Zhang et al. 2015), suggesting that increased feeding investment in provisioning females may proximately underlie elevated M_sum_. This interpretation assumes that M_sum_ is a reliable proxy for MMRs which is supported in some (e.g., Zhang et al. 2015), but not all cases (e.g., Wiersma et al. 2007). The fact that RMR and M_sum_ tended to correlate with different measures of reproductive investment suggests an uncoupling of these metabolic metrics that may reflect their divergent roles on organismal physiology and reproduction (Koteja 1987; Swanson et al. 2012; Careau et al. 2014). This is supported by the lack of correlations between RMR and M_sum_ (*r* = 0.06, *P* = 0.79; Supplementary Table S2; see Swanson et al. 2012; Careau et al. 2014).

In contrast with the results on metabolic traits, we did not find significant associations between CORT traits and reproductive output, with the exception of a trend for a positive association between BasCORT and reproduction (PC1) in one of our populations (MPG North). The latter association is consistent with the “CORT-adaptation” hypothesis (Chastel et al. 2005; Bonier et al. 2009, 2011), and probably related to the positive associations found between RMR and both PC1 (trend) and BasCORT. This interaction may however be interpreted with caution, given the small sample sizes after splitting our dataset by population. Not finding overall associations between CORT traits and components of reproduction is not necessarily surprising if metabolism mediates the effects of GCs on fitness, as that may lead to weaker relationships expected between CORT traits and reproduction, when compared to metabolic traits. Interestingly, a previous study showed that female violet-green swallows (*T. thalassina*) with experimentally increased energy expenditure (i.e., flight costs) during chick rearing also increased BasCORT, but had a similar reproductive output at the end of the season when compared to controls. Thus, GCs may help individuals cope with increased metabolic challenges during breeding and allow them to maintain fitness, which may mask the associations between GC concentrations and fitness components (Rivers et al. 2017). An alternative explanation for not finding significant associations between CORT and reproductive output may be that CORT is needed to reach higher metabolic rates, but also to obtain glucose from body reserves when resources are limited. Thus, the association between metabolic investment and CORT (and eventually, between CORT and fitness) may be masked or disrupted by resource availability (Jimeno et al. 2018c; Schoenle et al. 2018; Breuner and Berk 2019), which may differ between females and territories. Additionally, hormones respond rapidly to external or internal changes (Lema and Kitano 2013; Hau and Goymann 2015), and if the GC response in one context alters optimal endocrine phenotype in other contexts, these trade-offs may also shape the relationships between GCs and reproduction (Vitousek et al. 2018).

One limitation of our study is that we cannot test whether the metabolic and CORT traits that we measured at chick rearing, as well as feeding rate, reflect those traits at earlier or later stages of the breeding season. Trait repeatabilities may differ among seasons and years (Ouyang et al. 2011a; Vitousek et al. 2018), and previous research has shown that the change in CORT during the breeding season (i.e., from incubation to chick rearing), and not the absolute values, were predictors of reproductive output (Ouyang et al. 2011b). Thus, it is possible that this endocrine plasticity would have shown stronger or divergent correlations with reproductive outputs. Besides this, our limited sample size may have prevented us from detecting relationships (e.g., between CORT and metabolic or reproduction variables) for which the within-individual variability is relatively high when compared to the among-individual variability, or when trait variability is highly shaped by environmental factors. To this adds the fact that among-population and temporal variation strongly overlap in our dataset, which prevents us from making inferences regarding the factors driving population differences in metabolic and CORT trait values (Supplementary Fig. S4). Lastly, we did not monitor males in our study, which prevented us from accounting for potential adjustments between sexes when physiologically responding to reproductive demands.

Overall, our results show that metabolic traits are better predictors of reproductive output than CORT traits in breeding tree swallows, but that baseline GCs reflect individuals’ baseline metabolic investment during this period. Our findings further suggest that females with higher reproductive investment show higher upper limits of aerobic performance, together with greater energy expenditure at submaximal levels of activity.

## Data availability

Data are included as Supplementary Material.

## Supplementary Material

obaa030_Supplementary_Data
