# Supplementary material for: Metabolic Rates Predict Baseline Corticosterone and Reproductive Output in a Free-Living Passerine
Source: Integr Org Biol. 2020 Oct 14;2(1):obaa030. doi: 10.1093/iob/obaa030 (PMC7794023; doi:10.1093/iob/obaa030)
Supplement: obaa030_Supplementary_Data [file obaa030_supplementary_data.pdf]

SUPPLEMENTARY INFORMATION to:

Metabolic rates predict baseline corticosterone and reproductive output in a free-living passerine

Table S1: Sample size (number of females) available for each of the measurements included in the statistical models.

	**BasCORT**	**SICORT**	**RMR**	**M_{sum}**	**Scope**	**PC1**	**PC2**	**TOTAL**
N	23	24	24	24	22	18	18	**26**

Table S2: Pearson correlation matrix showing the correlations among metabolic traits, corticosterone traits, and reproduction variables. Correlation coefficients are presented in the lower diagonal, and p values in the upper diagonal. CORT variables were ln-transformed, and corticosterone and metabolic variables were standard-normalized by population to correct for between-population differences. Note that the strength of the associations and the p values of the correlation matrix may differ from those presented in the models, because models control by additional variables and they include principal components that integrate variables of reproduction. Significant ($p<0.05$) or close to significance ($p<0.09$) correlations appear in bold.

Metabolic and CORT traits were not correlated except for a trend between M_{sum} and SI-CORT after 10 minutes (r=-0.41, p=0.06). There were also no significant correlations within metabolic (RMR and M_{sum}; p>0.7) traits, with the exception of a high correlation between M_{sum} and aerobic scope, that are mathematically dependent. Baseline CORT was not correlated with stress-induced CORT (p=0.1), but showed high and negative correlations with stress-induced increases after 10 and 30 minutes (p<0.01). Stress-induced CORT levels after 10 and 30 minutes were positively correlated (p<0.03); stress-induced CORT after 30 minutes was positively correlated with CORT increase after that time (p=0.02), but there was no correlation between stress-induced CORT after 10 minutes and the associated increase (p=0.11). PHD 12-13 average chick mass was positively correlated with number of PHD 12-13 nestlings and number of hatchlings (p<0.01), whereas feeding rate was not correlated with any other variable of reproduction (p>0.6). Number of hatchlings and number of PHD 12-13 nestlings were also positively correlated (p<0.01), a correlation expected in a species with a high nestling survival rate.

	**BasCORT**	**SICORT_10**	**SICORT_30**	**Inc.10**	**Inc.30**	**RMR**	M_{sum}	**Scope**	**Chick mass**	**Feeds/h**	**Hatchlings**	**PHD13 ch.**
**BasCORT**		0.11	0.90	**<0.01**	**<0.01**	0.38	0.83	0.61	0.86	0.98	0.41	0.85
**SICORT_10**	0.34		**0.02**	0.11	0.54	0.76	**0.06**	0.09	**0.07**	0.68	**<0.01**	**0.08**
**SICORT_30**	-0.03	**0.46**		0.21	**0.02**	0.85	0.33	0.42	0.21	0.79	0.10	0.28
**Inc_10**	**-0.74**	0.34	0.27		**<0.01**	0.60	0.18	0.46	0.18	0.97	0.35	0.20
**Inc_30**	**-0.89**	-0.14	**0.47**	**0.77**		0.28	0.97	0.73	0.84	0.93	0.79	0.96
**RMR**	0.20	-0.07	-0.04	-0.12	-0.25		0.79	0.57	0.98	0.67	0.45	0.25
**M_{sum}**	-0.05	**-0.41**	-0.22	-0.30	-0.01	0.06		**<0.01**	0.89	0.44	0.21	0.68
**Scope**	-0.12	-0.38	-0.19	-0.18	0.08	-0.13	0.97		0.81	0.46	**0.07**	0.39
**Chick mass**	0.05	**0.47**	0.33	0.36	0.06	-0.01	-0.04	-0.06		0.95	**<0.01**	**<0.01**
**Feeds/h**	-0.01	0.09	0.06	-0.01	0.02	-0.10	-0.18	-0.17	-0.02		0.62	0.93
**Hatchlings**	0.19	**0.64**	0.36	0.21	-0.06	0.17	-0.28	**-0.40**	**0.92**	0.11		**<0.01**
**PHD13 ch.**	0.05	**0.45**	0.28	0.35	0.01	0.29	-0.11	-0.23	**0.89**	0.02	**0.92**	

p

R

Table S3: Model comparisons showing the best fitting models (△AICc<2) predicting stress-induced CORT (ln) after 10 (a) or 30 (b) min. of restraint, and including M_{sum} as predictor.

**(a) SI-CORT 10**	**K**	**logLik**	**AICc**	**△AICc**	**Weight**
M_{sum}	2	-13.89	35.1	0.00	0.45
	**Estimate**	**s.e.**	**d.f.**	**F**	**P**
Intercept	3.61	0.30	20 (1)		
M_{sum}	-0.17	0.05	20 (1)	10.64	<0.01
**(b) SI-CORT 30**	**K**	**logLik**	**AICc**	**△AICc**	**Weight**
Population	2	-8.53	24.4	0.00	0.32
Population, Body mass	3	-7.40	25.1	0.76	0.22
Population, M_{sum}	3	-4.59	25.5	1.15	0.18
	**Estimate**	**s.e.**	**d.f.**	**F**	**P**
Intercept	3.18	0.25	19 (1)		
Population	-1.01	0.21	19 (1)	24.14	<0.001
M_{sum}	-0.07	0.05	19 (1)	1.69	0.21

Table S4: Model comparisons showing the best fitting models (△AICc<2) predicting stress-induced increase in CORT after 10 (a) or 30 (b) min. of restraint, and including aerobic scope as predictor.

**(a) CORT increase 10**	**K**	**logLik**	**AICc**	**△AICc**	**Weight**
Population, Body mass	3	-7.90	26.7	0.00	0.24
Population	2	-9.78	27.2	0.50	0.19
Population, Scope	3	-8.23	27.3	0.66	0.17
Population, Scope, Body mass	4	-6.73	28.1	1.41	0.12
	**Estimate**	**s.e.**	**d.f.**	**F**	**P**
Intercept	1.40	0.24	15 (1)		
Population	0.94	0.21	15 (1)	19.99	<0.001
Body mass	0.14	0.08	15 (1)	2.58	0.13
Scope	-0.09	0.07	15 (1)	1.97	0.18
**(b) CORT increase 30**	**K**	**logLik**	**AICc**	**△AICc**	**Weight**
Population	2	-7.70	23.0	0.00	0.29
Population, Body mass	3	-6.62	24.1	1.10	0.17

Table S5: Model comparisons showing the best fitting models (△AICc<2) predicting reproductive output (PC1), and including Baseline CORT as predictor.

PC 1	K	logLik	AICc	△AICc	Weight
Null	1	-27.46	59.8	0.00	0.31
Population	2	-26.64	61.3	1.44	0.15
Population, BasCORT, BasCORT:Population	4	-22.79	61.6	1.73	0.13

	Estimate	s.e.	d.f.	F	P
Intercept	-10.69	3.82	13 (1)		
Population	11.02	3.86	13 (1)	8.14	0.01
BasCORT	5.32	1.96	13 (1)	7.36	0.02
BasCORT:Population	-5.26	2.01	13 (1)	6.82	0.02

Table S6: Model comparisons showing the best fitting models (△AICc<2) predicting reproductive output (PC1), and including stress-induced CORT after 10 (a) or 30 (b) min. Of restraint as predictors.

(a) PC 1	K	logLik	AICc	△AICc	Weight
Null	1	-28.66	62.2	0.00	0.32
Population	2	-27.88	63.6	1.41	0.16
**(b) PC 1**	**K**	**logLik**	**AICc**	**△AICc**	**Weight**
Null	1	-28.66	62.2	0.00	0.35
Population	2	-27.88	63.6	1.41	0.17

Table S7: Model comparisons showing the best fitting models (△AICc<2) reproductive output (PC1), and including stress-induced increase in CORT after 10 (a) or 30 (b) min. of restraint (b) as predictors. CORT increases were calculated as ln(stress-induced CORT) – ln(baseline CORT).

(a) PC 1	K	logLik	AICc	△AICc	Weight
Null	1	-28.66	62.2	0.00	0.36
Population	2	-27.88	63.6	1.41	0.18
**(b) PC 1**	**K**	**logLik**	**AICc**	**△AICc**	**Weight**
Null	1	-28.66	62.2	0.00	0.36
Population	2	-27.88	63.6	1.41	0.18

Table S8: Model comparisons showing the best fitting models (△AICc<2) reproductive output (PC1), and including M_{sum} (a) or aerobic scope (b) as predictors.

(a) PC 1	K	logLik	AICc	△AICc	Weight
Null	1	-29.07	63.0	0.00	0.33
Population	2	-27.95	63.7	0.76	0.23
**(b) PC 1**	**K**	**logLik**	**AICc**	**△AICc**	**Weight**
Null	1	-27.46	59.8	0.00	0.39
Population	2	-26.64	61.3	1.44	0.19

Table S9: Model comparisons showing the best fitting models (△AICc<2) reproductive output (PC2), and including RMR as predictor.

PC 2	K	logLik	AICc	△AICc	Weight
Null	1	-24.20	53.3	0.00	0.42

Table S10: Model comparisons showing the best fitting models (△AICc<2) reproductive output (PC2), and including Baseline CORT as predictor.

PC 2	K	logLik	AICc	△AICc	Weight
Null	1	-22.46	49.8	0.00	0.44

Table S11: Model comparisons showing the best fitting models (△AICc<2) reproductive output (PC2), and including stress-induced CORT after 10 (a) or 30 (b) min. of restraint (b) as predictors.

(a) PC 2	K	logLik	AICc	△AICc	Weight
Null	1	-24.20	53.3	0.00	0.37
SI-CORT 10 (ln)	2	-23.64	55.1	1.85	0.15
	**Estimate**	**s.e.**	**d.f.**	**F**	**p**
Intercept	1.16	1.24	15 (1)		
SI-CORT 10 (ln)	-0.45	0.45	15 (1)	1.04	0.32
**(b) PC 2**	**K**	**logLik**	**AICc**	**△AICc**	**Weight**
Null	1	-24.20	53.3	0.00	0.39

Table S12: Model comparisons showing the best fitting models (△AICc<2) reproductive output (PC2), and including stress-induced increase in CORT after 10 (a) or 30 (b) min. of restraint (b) as predictors. CORT increases were calculated as ln(stress-induced CORT) – ln(baseline CORT).

(a) PC 2	K	logLik	AICc	△AICc	Weight
Null	1	-24.20	53.3	0.00	0.41
**(b) PC 2**	**K**	**logLik**	**AICc**	**△AICc**	**Weight**
Null	1	-24.20	53.3	0.00	0.41

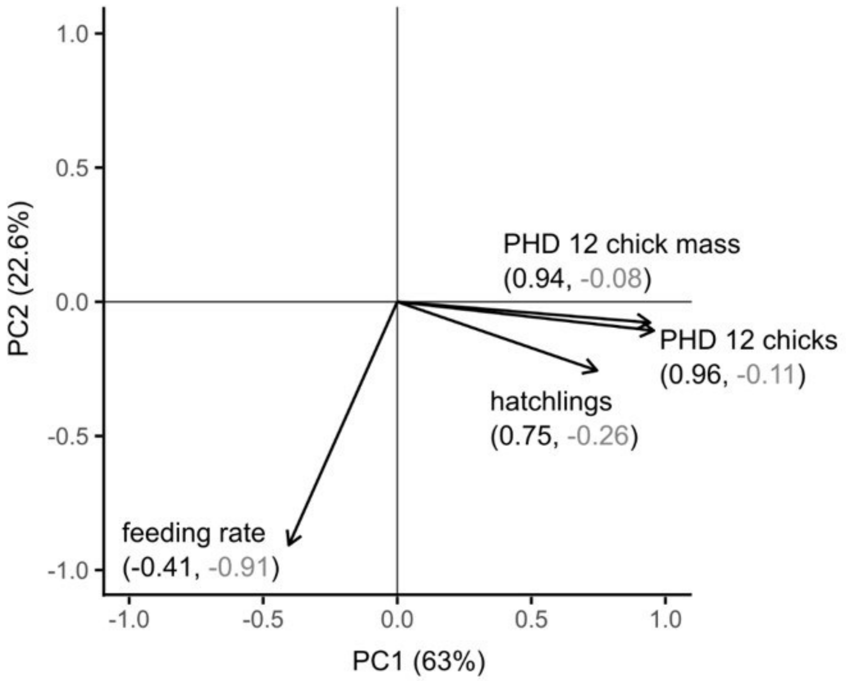

Fig. S1: Results of the PC analysis on reproductive output (two main components). Factor loadings for PC1 are presented in black, whereas factor loadings for PC2 are presented in grey. Note that the sign of PC2 (which originally had negative values for higher feeding rates) was changed in the text and in Fig. 3 for clarity and interpretation reasons, so that higher PC2 values are associated with higher feeding rates. The following reproductive output variables were included: hatchlings (number of hatchlings), PHD 12 chicks (number of nestlings at PHD 12-13), PHD 12 chick mass (average PHD 12-13 nestling mass), feeding rate (number of female feeds / hour, PHD 10-12).

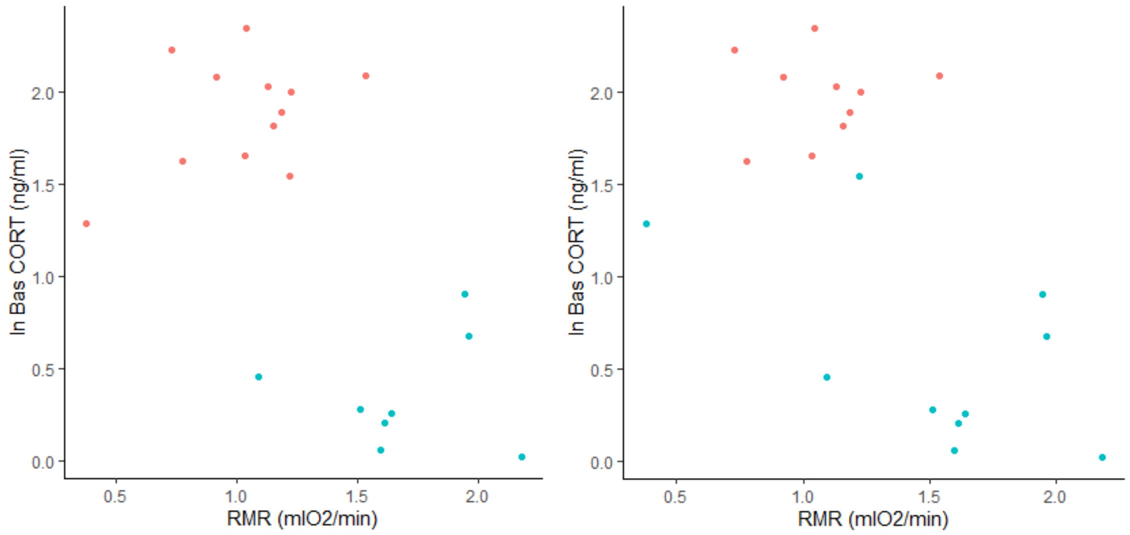

Fig. S2. Correlation between RMR and Baseline CORT by population (left panel) or year (right panel). Different colours represent the two populations (Red: MPG North; Blue: MPG Ranch) and years (Red: 2016; Blue: 2017).

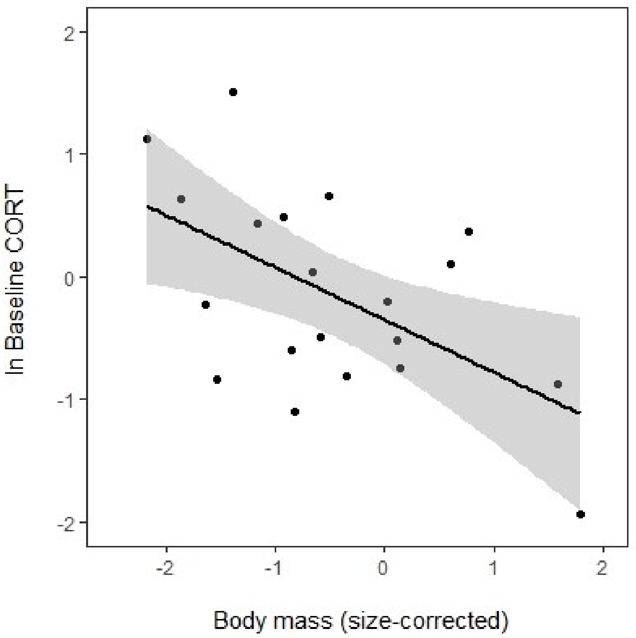

Fig. S3: Relationship between baseline CORT and residual body mass (corrected by structural size). Note that Baseline CORT (ln) was standard-normalized by population (x-mean/ sd) for visualization purposes.

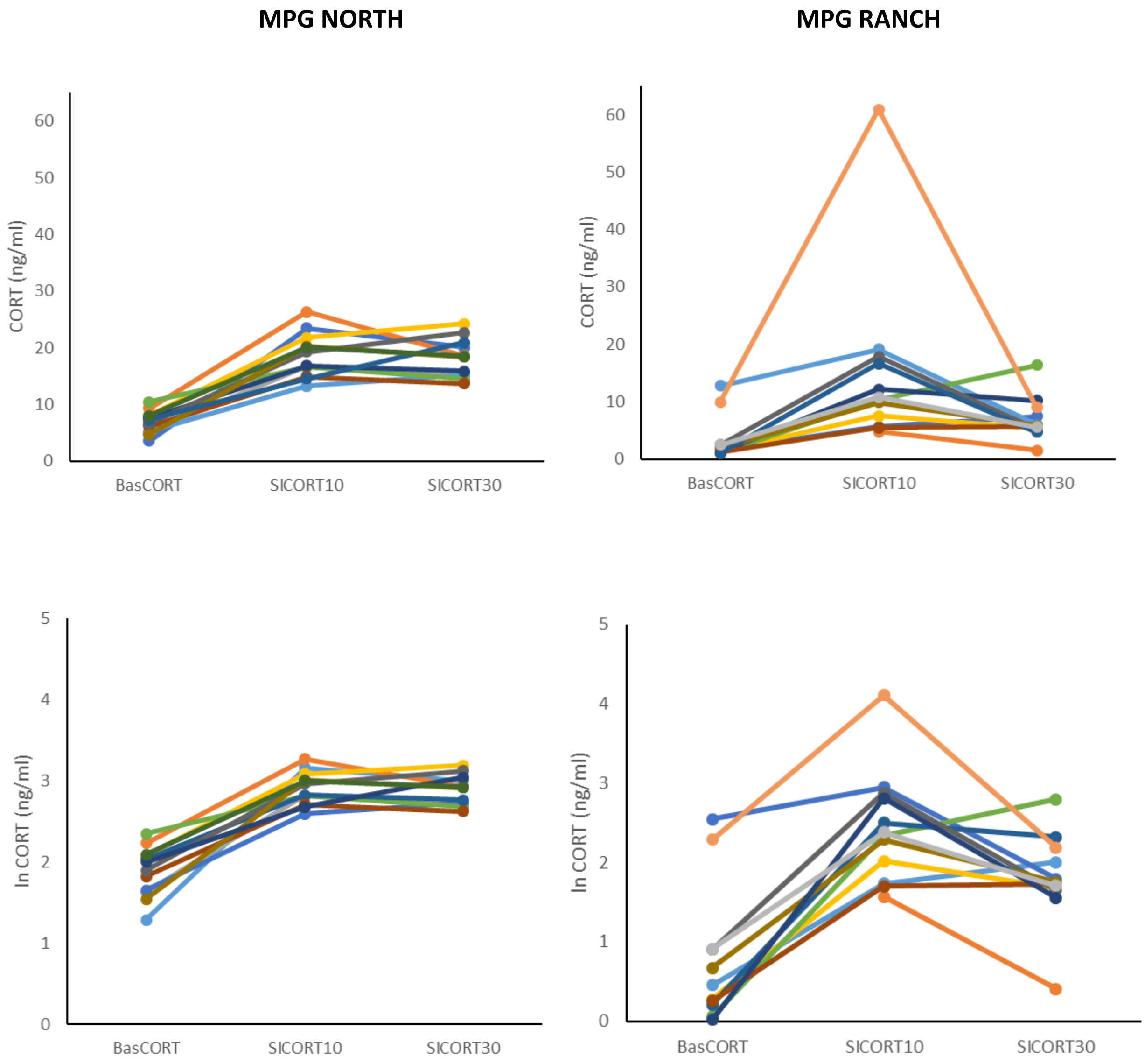

Fig. S4: Individual CORT values (baseline, stress-induced after 10 minutes of restraint, and stress-induced after 30 minutes of restraint) by population. The graphs show absolute CORT values for the two populations included in the study (upper panels), as well as ln-transformed values, as included in the models (lower panels).
